# Co-formulation of the rF1V plague vaccine with depot-formulated cytokines enhances immunogenicity and efficacy to elicit protective responses against aerosol challenge in mice

**DOI:** 10.3389/fimmu.2024.1277526

**Published:** 2024-03-28

**Authors:** Darrell R. Galloway, Jiahui Li, Nguyen X. Nguyen, Frank W. Falkenberg, Lisa Henning, Robert Krile, Ying-Liang Chou, James N. Herron, J. Scott Hale, E. Diane Williamson

**Affiliations:** ^1^ Department of Molecular Pharmaceutics, University of Utah, Salt Lake City, UT, United States; ^2^ Department of Pathology, Division of Microbiology and Immunology, University of Utah, Salt Lake City, UT, United States; ^3^ CIRES, GmbH, Bochum, Germany; ^4^ CyTuVax BV, Maastricht, Netherlands; ^5^ Battelle Biomedical Research Center, Columbus, OH, United States; ^6^ Chemical Biological Radiological Division, Defense Science and Technology Laboratory (DSTL), Porton Down, Salisbury, United Kingdom

**Keywords:** vaccination, plague vaccine, cytokine depot adjuvant, *Yersinia pestis*, subunit vaccine, co-immunization, immune enhancement, protective antibody titer

## Abstract

This study evaluated a depot-formulated cytokine-based adjuvant to improve the efficacy of the recombinant F1V (rF1V) plague vaccine and examined the protective response following aerosol challenge in a murine model. The results of this study showed that co-formulation of the Alhydrogel-adsorbed rF1V plague fusion vaccine with the depot-formulated cytokines recombinant human interleukin 2 (rhuIL-2) and/or recombinant murine granulocyte macrophage colony-stimulating factor (rmGM-CSF) significantly enhances immunogenicity and significant protection at lower antigen doses against a lethal aerosol challenge. These results provide additional support for the co-application of the depot-formulated IL-2 and/or GM-CSF cytokines to enhance vaccine efficacy.

## 1 Introduction

In spite of more than a century of effort, plague remains one of the most feared infectious diseases known to mankind (1). Plague, which remains endemic to this day, manifests itself in two clinical forms, i.e., bubonic and pneumonic, both of which are extremely infectious (1). *Yersinia pestis*, which infects numerous animal hosts, including rodents, is the causative organism (2). An intermediate host is the common flea, which has been demonstrated to make humans an incidental host (3). The result has been a series of pandemics that changed the course of human history (2, 4). A recent outbreak in Madagascar involved more than 2,500 cases with a fatality rate of 8.6%, which serves as a reminder that this disease remains a threat (5). An additional concern is the reminder that plague has previously been used as a biological weapon and remains a concern in that regard (6). For these reasons, the WHO has issued a call and guidelines for the development of an effective vaccine against plague (7).

The study of plague has involved the use of several animal models (8, 9). The results of these studies have demonstrated that two antigens appear to be the optimum targets for the development of a protective immune response against aerosolized plague. Specifically, the F1 capsular antigen (10) and the LcrV antigen, which is associated with the low calcium response in *Y. pestis* (11–14), appear to be sufficient for complete protection against aerosolized plague. It has previously been shown that vaccination with the F1 antigen alone, while protective, is not sufficient to provide protection against virulent F1-negative strains of *Y. pestis* in aerosol challenge studies. Therefore, a lot of the vaccine studies have concentrated on a single recombinant F1V fusion protein that includes both antigens (15, 16) and vaccines containing both the F1 and LcrV antigens (17–19) in order to provide complete protection. It has been shown that immunization with a combination of the recombinant F1 (rF1) and recombinant LcrV (rLcrV) antigens elicits a greater degree of protection compared with vaccination with rF1 or rLcrV alone (20).

Despite years of effort, a licensed plague vaccine that protects against aerosolized plague is not yet available. The most advanced plague vaccines are those produced in the USA [consisting of a recombinant F1V (rF1V) fusion protein formulated with Alhydrogel—alum or aluminum hydroxide wet gel suspension] and the UK (consisting of rF1 and rLcrV formulated with Alhydrogel). The American rF1V vaccine was withdrawn from phase 2b clinical studies due to its lack of efficacy (low immunogenicity and lack of memory response). The two-component (rF1 + LcrV) vaccine has also undergone evaluation in clinical studies (17); however, it is not currently in advanced development.

There have been numerous studies carried out in an effort to improve the immune response against the F1 and LcrV antigens (21–24). The use of cytokines as immune adjuvants has been widely studied, and previous studies have revealed that interleukin 2 (IL-2) and granulocyte macrophage colony-stimulating factor (GM-CSF) enhance the production of antibodies against various protein antigens (25–29). The current study describes the novel application of these cytokines formulated to result in the delivery of highly concentrated forms of these molecules to the lymph nodes (30).

The objective of this study was to evaluate Alhydrogel-adsorbed cytokines (termed cytokine depots) with respect to the immunogenicity and efficacy of the rF1V vaccine.

## 2 Materials and methods

### 2.1 Animals, immunizations, and challenges

#### 2.1.1 Plague vaccine formulations

The purified vaccine antigen, rF1V, was formulated with Alhydrogel (InvivoGen, San Diego, CA, USA) and various Alhydrogel-adsorbed cytokine combinations to determine whether the cytokine depot adjuvants confer enhanced protection following a two-immunization sequence (days 0 and 21) using the subcutaneous (s.c.) route. The rF1V antigen was adsorbed to Alhydrogel at a 1:3 wt/wt ratio to ensure complete binding of the rF1V antigen. The cytokine depot adjuvants consisted of either recombinant human IL-2 (rhuIL-2) or recombinant murine GM-CSF (rmGM-CSF) (PeproTech, Cranbury, NJ, USA), or both, adsorbed to an excess of Alhydrogel to ensure complete binding of all the cytokines. The Alhydrogel-adsorbed rF1V was co-administered with the Alhydrogel-adsorbed cytokines just prior to immunization using a final volume of 0.1 mL. **Table 1** outlines the four different vaccine groups, including a phosphate-buffered saline (PBS) vaccine control group. The vaccine composition per dose (0.1 mL) for each group is also shown in the table.

**Table 1:** Summary of the different vaccine groups, composition of the vaccine formulations, and the aerosol protection results.

Group	No. of mice	Vaccine	Antigen (µg/dose)	Adjuvant	Adjuvant (µg/dose)	First	Second	No. of deaths	% survival
1	10	rF1V fusion	10	Alhydrogel	30	SC	SC	1	90
2	10		1		3			4	60
3	10		0.1		0.3			10	0
4	10	rF1V fusion	10 µg F1V + 30 µg Alhydrogel	IL-2	10 µg IL-2 + 10 µg Alhydrogel	SC	SC	0	100
5	10		10 µg F1V + 30 µg Alhydrogel	GM-CSF	10 µg GM-CSF + 10 µg Alhydrogel			0	100
6	10		10 µg F1V + 30 µg Alhydrogel	IL-2 and GM-CSF	IL-2 + 10 µg Alhydrogel; 10 µg GM-CSF + 10 µg Alhydrogel			0	100
7	10		1 µg F1V + 3 µg Alhydrogel	IL-2	10 µg IL-2 + 10 µg Alhydrogel			2	80
8	10		1 µg F1V + 3 µg Alhydrogel	GM-CSF	10 µg GM-CSF + 10 µg Alhydrogel			1	90
9	10		1 µg F1V + 3 µg Alhydrogel	IL-2 and GM-CSF	IL-2 + 10 µg Alhydrogel; 10 µg GM-CSF + 10 µg Alhydrogel			0	100
10	15	PBS	N/A	N/A	N/A	SC	SC	15	0

rF1V, recombinant plague vaccine; GM-CSF, granulocyte macrophage colony-stimulating factor; PBS, phosphate-buffered saline; N/A, not applicable; SC, sub-cutaneous. IL-2, Interleukin-2.

#### 2.1.2 Animal immunization

All animal immunization and challenge studies were carried out at the Battelle Biomedical Research Center, Columbus, OH, USA, under an NIH contract (NIAID’s Preclinical Services contract no. HHSN272201200003I/HHSN27200027) and previously approved by the Institutional Animal Care and Use Committee (IACUC). The vaccine groups consisted of 10 Balb/c mice (Charles River Laboratories, Wilmington, MA, USA) 8–12 weeks of age (50% males/50% females). The mice were identified by Labstamp^®^ tail tattoos and were also implanted with temperature transponders to monitor body temperature during the course of the study (s.c.; IPTT-300, BMDS, Waterford, WI, USA). On day 0, the mice received s.c. vaccinations in the flanks with varying doses of antigen (rF1V fusion protein) adsorbed to Alhydrogel or co-administration of cytokines (IL-2/Alhydrogel and/or GM-CSF/Alhydrogel). On day 21, the mice were boosted *via* the s.c. route with the corresponding vaccine. The control group was administered PBS (s.c.) on days 0 and 21. The injection sites were observed for adverse reactions, including erythema (redness) and edema (swelling), twice daily.

#### 2.1.3 Aerosol challenge

At the end of the vaccination series (day 42), the mice were challenged *via* the aerosol route using a suspension of *Y. pestis* CO92 in PBS + 0.01% gelatin with 9.7% α-α-trehalose (BSGT) under BSL3 conditions using a *Y. pestis* dose of 1.5 × 10^5^ colony forming units (CFU)/mouse. Challenge was performed as previously described (23). Following the challenge, all animals were monitored for a period of 14 days for the onset of clinical signs. Any animals displaying clinical signs such as weight loss or ruffled fur were euthanized as prescribed by the IACUC-approved humane end point. All animals were euthanized 14 days following the challenge. Spleen homogenates were plated onto CIN agar to evaluate *Y. pestis* tissue burden. A complete gross necropsy was performed on all mice.

### 2.2 Reagents

The purified recombinant F1V fusion protein was the generous gift of the U.S. Defense Department and was provided through a cooperative research agreement with the Joint Program Executive Office (JPEO). The recombinant human IL-2 rhuIL-2 and rmGM-CSF were purchased from PeproTech. The Alhydrogel [Alhydrogel “85” (2% suspension)] aluminum hydroxide gel adjuvant was purchased from Brenntag Biosector A/S, 3600 (Frederikssund, Denmark).

### 2.3 Enzyme-linked immunosorbent assay

Serum from all immunized animals was used for the determination of an rF1V antibody titer for comparative purposes. Blood was collected by mandibular puncture on days 0, 14, 35, and 42 of the study. The serum from individual mice was analyzed for anti-rF1V immunoglobulin G (IgG) using ELISA as previously described in detail (19, 31). For comparative analysis, titers were calculated as micrograms of the anti-F1V antibody per milliliter of serum.

### 2.4 Statistical analysis

A variety of statistical tools were used in the analysis of the data from this study. Exact binomial confidence intervals were used to determine the survival rates, while exact 95% confidence intervals for each dosage were calculated. To determine whether the survival outcome for each group was superior to that of the control group, the one-sided Boschloo’s exact test was used. To determine whether there were any differences in protection in the groups based on a time to death model, we compared the time to death data with the survival data using Kaplan–Meier curves and a log-rank test. In order to maintain an overall 0.05 significance level, the Bonferroni–Holm adjustment for multiple comparisons was used. In this study, both the unadjusted and multiple comparison-adjusted outcomes were compared.

## 3 Results

### 3.1 Co-administration of rF1V bound to Alhydrogel with IL-2 and GM-CSF adsorbed to Alhydrogel enhances protection against aerosol challenge in BALB/c mice in a dose-dependent manner

The study started with a comparison of the immunogenicity and efficacy of the rF1V vaccine formulated with IL-2 and/or GM-CSF adsorbed to Alhydrogel in a mouse model of pneumonic plague. **Table 1** provides an outline of the study, showing the different vaccine groups, the composition of the vaccine formulations, and a summary of the aerosol protection results. Each vaccination consisted of a mixture of the rF1V antigen and the cytokine depot adjuvant in an injection volume of 0.1 mL. It is to be noted that an additional series of challenges were carried out using the rF1 and LcrV antigens, with similar results (data not shown).

The rF1V fusion vaccines were efficacious against the *Y. pestis* challenge in a dose-dependent manner. The groups administered the rF1V fusion vaccine had significantly greater survival rates and times to death than the PBS control group. Specifically, the administration of 10, 1, and 0.1 μg rF1V fusion antigen adsorbed to 30, 3, and 0.3 μg Alhydrogel resulted in 90%, 60%, and 0% survival, respectively. The administration of all high-dose (10 μg) rF1V fusion cytokine groups resulted in 100% survival, while the administration of the low-dose (1 μg) rF1V cytokine groups resulted in 80% (IL-2), 90% (GM-CSF), and 100% (IL-2/GM-CSF) survival. Survival at the lower dose range (a 1:10 dilution) using the cytokine adjuvant co-administered with the rF1V vaccine is suggestive of the increased efficacy of the cytokine adjuvant co-formulated vaccine.

There were no adverse reactions observed during the post-vaccination period, as measured by visual observations, body temperature, and body weight. While the group mean body temperatures remained relatively consistent during the time points measured in the post-vaccination period, most groups had significant increases in body temperature when the post-vaccination time points were compared with the baseline (prior to vaccination), including the PBS control group. In addition, the group mean body weights increased over time during the post-vaccination period. For all study groups, the mean body weights on days 14, 35, and 49 were significantly higher than those at the baseline at the 0.05 level. Abnormal injection site reactions (edema and erythema) were observed in all groups, including the PBS control. Therefore, the observed edema and erythema were related to the administration site.

### 3.2 Co-administration of the rF1V vaccine with IL-2 and GM-CSF bound to Alhydrogel enhances anti-F1V antibody response

**Figure 1:**
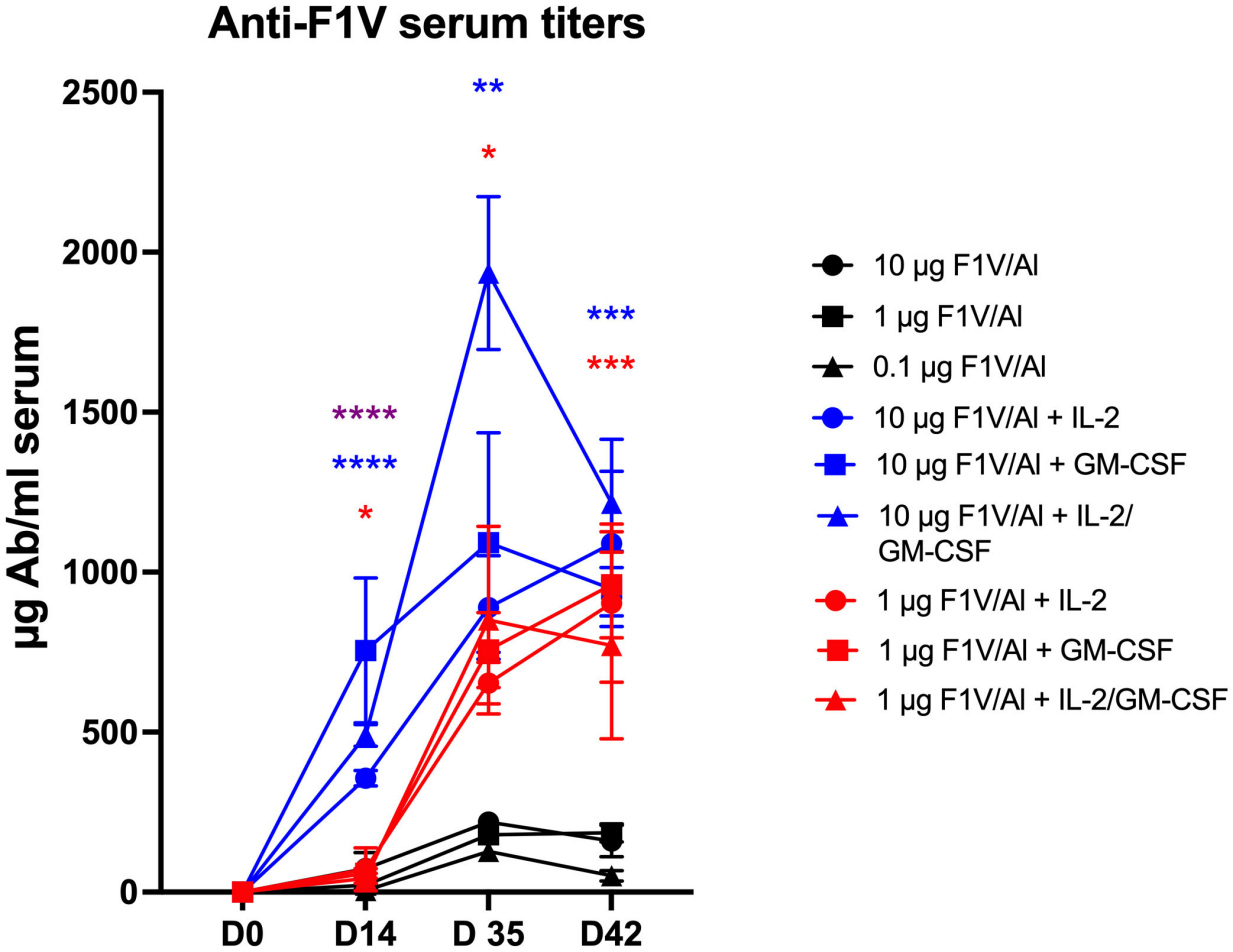
Anti-F1V serum titers. Time course of the anti-F1V responses comparing Alhydrogel and the cytokine co-formulated vaccines. Statistically significant p values were determined using a two-tailed unpaired Student t test; *p < 0.05, **p < 0.01, ***p < 0.001, ****p < 0.0001.

**Figure 1** shows that the addition of Alhydrogel-adsorbed cytokines (rhuIL-2 and/or GM-CSF) to Alhydrogel-adsorbed rF1V significantly enhanced the specific anti-F1V antibody titer with respect to both time and magnitude when compared with the response following immunization with rF1V formulated with Alhydrogel alone. At a higher dose of rF1V (10 µg), the initial response (day 14) following a single immunization with cytokines was higher and fell within a protective range based on the results of a previously published report (23). Even a lower dose of 1 µg rF1V combined with cytokines resulted in a significant response following the second immunization when compared with the response following a second immunization with the rF1V Alhydrogel-only formulation. Thus, the cytokine adjuvant induces a higher anti-F1V response in a shorter period when compared with the F1V Alhydrogel-only group.

### 3.3 Co-administration of rF1V with cytokines extends the time to death response in a dose-dependent manner

**Figure 2:**
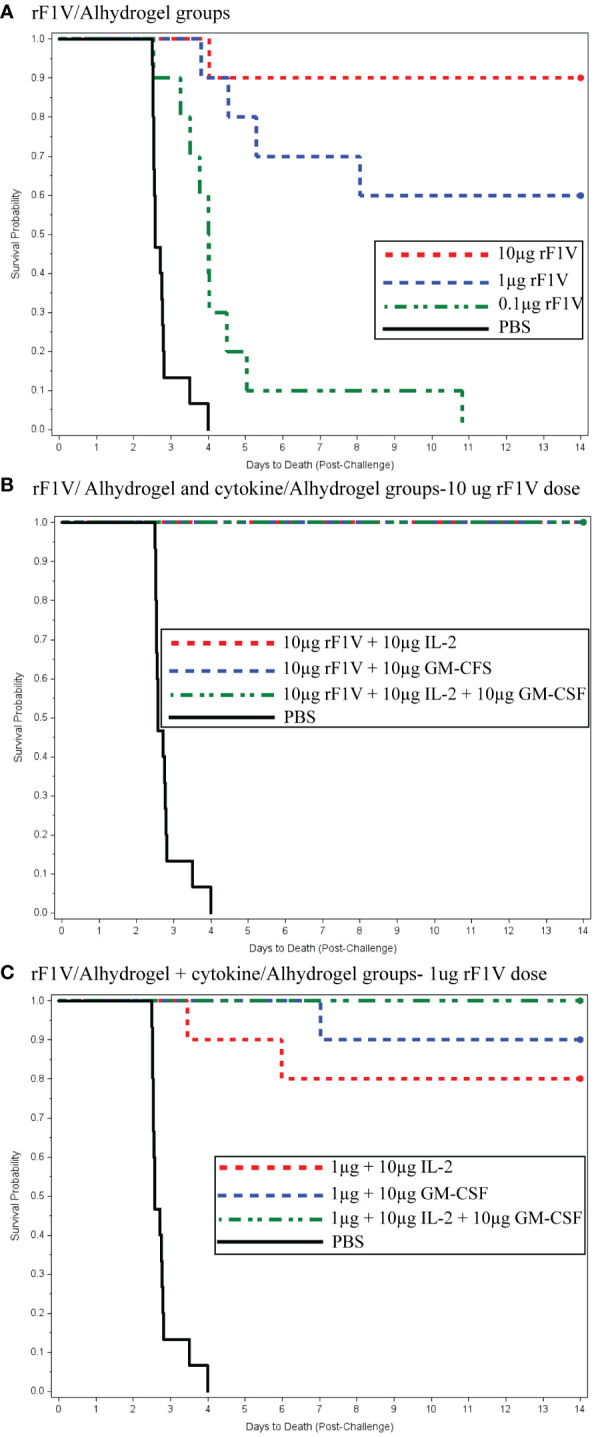
**(A)** Recombinant F1V (rF1V)/Alhydrogel groups. **(B)** rF1V/Alhydrogel and cytokine/Alhydrogel groups—10 µg rF1V dose. **(C)** rF1V/Alhydrogel + cytokine/Alhydrogel groups—1 µg rF1V dose.

**Figures 2A–C** display the Kaplan–Meier time to death comparisons between the different vaccine groups, which demonstrated that the mean time to death was significantly enhanced in a dose-dependent manner by the co-administration of the rF1V plague vaccine with cytokine adjuvants.

In **Figure 2A**, for the F1V Alhydrogel groups (10, 1, and 0.1 μg), logistic regression models were fitted to the survival data as a function of the base 10 logarithms of vaccine dose. The likelihood ratio test (LRT) was statistically significant at the 0.05 level, indicating a significant relationship between survival and the vaccination dose. The estimated vaccine doses for the 50th and 90th survival percentiles were 1.00 and 16.62 µg, respectively. **Figure 2B** shows the survival rates for the high-dose F1V cytokine groups, all of which showed 100% survival. **Figure 2C** presents the time to death plot for the low-dose F1V cytokine group. Administration of 1 µg rF1V fusion/3 µg Alhydrogel + 10 µg IL-2/10 µg Alhydrogel, 10 µg GM-CSF/10 µg Alhydrogel, and 10 µg IL-2/10 µg GM-CSF/10 µg Alhydrogel resulted in 80%, 90%, and 100% survival, respectively. Both prior to and after adjusting for multiple comparisons, the low-dose F1V cytokine groups had significantly greater survival rates than the PBS control (**Figure 2C**). Similarly, both prior to and after adjusting for multiple comparisons, the times to death in the low-dose F1V cytokine groups were significantly greater than that in the PBS control group. Statistical analysis revealed that there were no statistically significant differences in the survival rates between the respective high- and low-dose cytokine F1V fusion groups. Both prior to and after adjusting for multiple comparisons, there were no statistically significant differences in time to death between the high-dose and low-dose cytokine groups.

### 3.4 Co-immunization with cytokines adsorbed to Alhydrogel enhances the protective response by reducing the bacterial burden

**Figure 3:**
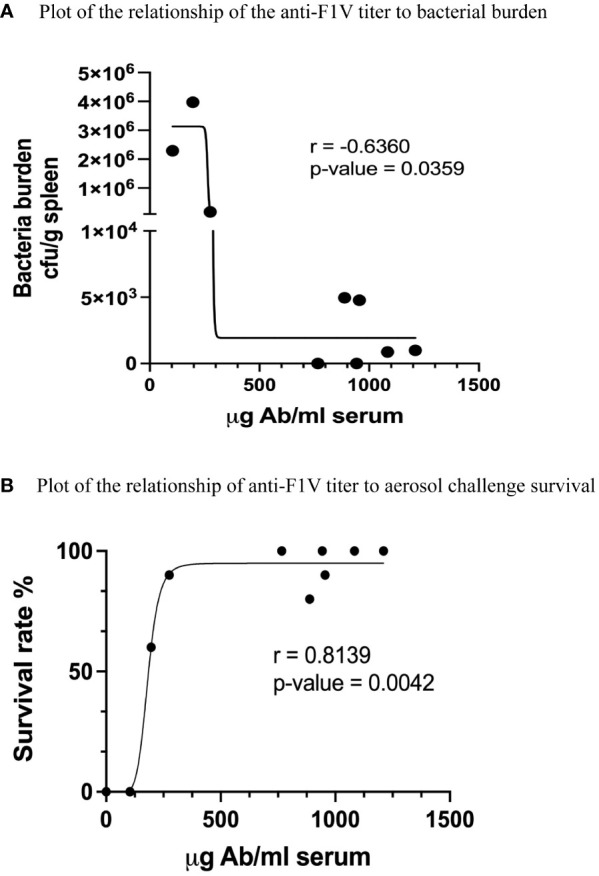
Analysis of the results comparing **(A)** bacterial burden and **(B)** anti-F1V- specific titers to overall survival in aerosol-challenged mice, using linear regression fit of the data. Spearman correlations (r values) were calculated using the GraphPad Prism software program.

Two weeks after the aerosol challenge, all surviving mice were euthanized and the spleen of all the animals used in the study cultured for the presence of *Y. pestis* in order to determine the extent, if any, of bacterial burden on the outcome. The PBS control mice showed a significant bacterial burden (>10^8^ CFU/g) from the spleen cultures compared with vaccinated mice (10^5^–10^0^ CFU/g). The results are depicted in **Figure 3A**, where a significant difference in the bacterial burden between the non-adjuvanted and the cytokine-adjuvanted groups can be observed. Both the high-dose (10 μg F1V) and the low-dose (1 μg F1V) adjuvanted groups displayed a markedly reduced bacterial burden when compared with the high- (10 μg) and low-dose (1 μg) non-adjuvanted groups. These results appear to correlate with the survival rate, demonstrating that the co-administration of cytokine adjuvants is correlated with increased protection. **Figure 3B** compares the anti-F1V titers with the outcome of the aerosol challenge. A distinct difference in the degree of protection based on the level of anti-F1V titer was observed. Taken together, these results suggest that the co-administration of cytokine depot adjuvants contributes to survival by reducing the bacterial burden, thus reducing the spread of infection.

## 4 Discussion

The results presented in this study demonstrated that co-formulation of the rF1V vaccine with Alhydrogel in combination with the Alhydrogel-bound adjuvant cytokines IL-2 and GM-CSF, or both, significantly enhances both the immunogenicity and efficacy and the protection against a significant aerosol challenge with *Y. pestis* in a well-accepted murine model. The results of this study are a logical follow-on to the findings presented in our previous paper (31), which demonstrated that cytokine depot adjuvants induced key reactions between germinal center B cells and CD4^+^ T cells through the T follicular helper (Tfh) system, resulting in both higher anti-F1V titers and the induction of a long-term immune response to F1V. These results included an analysis of the rF1 and LcrV antigens and were predictive of the results reported in this study showing enhanced protection in a significant aerosol challenge model. The results of this study are also consistent with the levels of anti-F1V antibody that were determined to be predictive of a >90% level of protection as reported by Moore et al. (23), who examined both the rF1V and the two-component rF1+rLcrV vaccines using the same aerosol challenge model (23).

Co-administration of particulate cytokine depot formulations is a novel approach to vaccine enhancement and demonstrates the potential for application to the enhancement of other vaccines. Adjuvants have long been used to enhance the magnitude and length of the immune response; however, complete understanding of their mode(s) of action remains elusive. In recent years, a more detailed understanding of the mechanisms of action of adjuvants has resulted from a better elucidation of the role of the innate immune system and its subsequent induction of the adaptive immune response. Understanding the relationship between the innate and adaptive immune responses has been one of the key reasons behind the emergence of new adjuvants, e.g., MF59, AS01, AS03, AS04, and CpG 1018 [reviewed in (32–34)]. In particular, the identification of cellular receptors that recognize the various pathogen molecules present in many cells has been a key factor. These include the Toll-like receptors (TLRs), which are often found on dendritic cells (DCs). Pathogen-associated molecules that bind to these receptors (pathogen-associated molecular patterns, PAMPs) result in the activation of DCs, which leads to the stimulation of various antigen-specific B and T cells. Following immunization, DCs that have been activated by localized adjuvants at the injection site take up the localized antigens and later present these antigens to naive antigen-specific CD4^+^ T helper cells in the lymphoid organs. Within the lymph node, the activated T helper cells stimulate the clonal expansion of the antigen-activated B cells into short-lived plasma cells or, within the germinal centers (GCs), into antigen-specific memory cells or long-lasting plasma cells (LLPCs) through a series of interactions involving Tfh cells (35–37). These interactions, which are crucial to the development of long-term memory B cells, are enhanced by various cytokines. Clearly, one of the strategies to enhance the induction of antigen-specific memory B cells is the specific targeting of the Tfh system using adjuvants. We have recently completed studies supporting this concept (31).

The principal approved adjuvant in most vaccines consisted of formulations with hydrated insoluble aluminum salts (Alhydrogel, alum), which have been in general use for more than seven decades. Alhydrogel has been a key component of the rF1V plague vaccine formulations in both experimental preclinical and clinical studies (15–18, 20). Vaccine formulation with aluminum-based adjuvants has recently been reviewed in some detail (38–42), with some general observations applicable to the findings reported in this study. In this study, we described an optimized use of alum to deliver functional cytokine molecules to the lymph nodes, which resulted in an enhanced immune response when co-administered with the immunogenic antigen, in this case the F1V fusion protein.

The effects of alum on immune enhancement have often been attributed to a “depot effect” that results in the slow release of antigens attached to the hydrated alum matrix (41, 42) from the inoculation site. The prolonged immunogen bioavailability is thought to result in the continuous stimulation of the immune reaction within the lymph node GCs (41). However, the concept of a depot effect for aluminum hydroxide has been questioned in a number of studies, implying that Alhydrogel elicits additional effects on the immune response not associated with its particulate nature (43–46). For example, there are reports that alum activates the NLR3P inflammasome (47, 48). However, recent evidence in mice deficient in NLR3P signaling has provided conflicting results that question whether activation of the NLR3P inflammasome is required for the adaptive immune response (49). Furthermore, numerous studies have reported that alum-induced cellular damage and the release of cellular components, including DNA, are responsible for a number of effects on the immune response (50, 51). Clearly, the use of Alhydrogel results in diverse stimuli of both the innate and adaptive immune responses, beginning with the recruitment and activation of the DCs at the inoculation site (44, 45). It seems reasonable to assume a likely role in stabilizing the antigen at the inoculation site and beginning the process of transfer to the regional lymph nodes where fragments of the antigen are presented with T cells to help naive B cells, ultimately resulting in an antigen-specific adaptive response.

The unique aspect of the present study is specifically associated with the co-administration of Alhydrogel-adsorbed cytokines (IL-2 and GM-CSF) to enhance the vaccine’s immunogenicity and efficacy in conjunction with a defined recombinant protein vaccine (rF1V). This study, as well as the results obtained, differs from other cytokine adjuvant studies in two substantial ways. Firstly, most studies using IL-2 and/or GM-CSF as adjuvants have been associated with cancer vaccines and influences on the outcomes in various cancer models (human or animal) (25–27). The use of such complex models involving complex systems has made it difficult to determine whether the use of these cytokine adjuvants is useful. In most of the cases reported, it is not. A second major and significant difference is that previous studies have applied the cytokines in a continuous or repeated manner during the course of vaccination (28, 29). As such, the biodistribution of these cytokines varies over time and is subject to dilution. In the present study, the two cytokines were adsorbed to an Alhydrogel vehicle and appear to be released locally, in discrete packets in a highly concentrated form (30). It is also suspected that these cytokine “particles” reach the lymph nodes where they are in close proximity to the B and T cells within the GCs. Therefore, the adjuvant properties of IL-2 and GM-CSF are largely within a confined microenvironment, where they influence the expression of various cytokines and other regulatory molecules in tandem with the co-administered antigen rF1V to regulate cellular interactions within the lymph node. It seems clear that this co-administration produces a unique and enhanced spatial–temporal stimulation of T cells by cytokines, IL-2 in particular, improving protection based on antigen presentation alone.

Oyler-Yaniv et al. (30) developed mathematical and experimental models that illustrate how IL-2-requiring cells arrange themselves in the proximity of IL-2-donating cells in order to bind a number of IL-2 molecules. These interactions take place in the 100-µm range around an IL-2-donating cell. Assen and Sixt have pointed out the importance of being able to consider and target these cell-to-cell interactions between the IL-2 donor and IL-2 recipient cells and their functions in the lymph node (52).

In support of this cell presentation concept, it has been shown that daily i.v. injections of IL-2 alone or of IL-2 and anti-IL-2 antibodies induce a 100-fold enhanced expansion of CD8^+^ T cells in the lymph node (53). In addition, this study demonstrated that injection of IL-4/anti-IL-4 complexes also leads to extremely high proliferation of CD8^+^ T cells. Similar results were also reported for IL-7. Tomala et al. (54) showed that the administration of IL-2/anti-IL-2 complexes results in similar stimulation in CD8^+^ T cells in the lymph nodes. IL-2/anti-IL-2 complexes provided better results than IL-2 alone, most probably due to the fact that the antibody-bound IL-2 is protected from the loss of IL-2 by diffusion and by protection of IL-2 from degradation. Tomala et al. cited several studies in which such protective effects have also been reported for IL-3, IL-4, IL-6, IL-7, and GM-CSF when they were injected as complexes together with their corresponding antibodies. These reports clearly demonstrate a role for concentrated, localized IL-2 presentation in the lymph node microenvironment. Similar observations have been reported by Falkenberg et al. (55) in experiments in which IL-2 adsorbed to alum was co-administered with an alum-bound vaccine target antigen for immunization, showing a correlation between the concentration of the alum-bound IL-2 and higher antibody response.

The results reported in this study suggest that vaccine antigen presentation within the lymph node microenvironment to elicit host protection is enhanced by co-administration with Alhydrogel-adsorbed cytokines. We have recently published studies indicating that the simultaneous presentation of rF1V in the presence of both IL-2 and GM-CSF cytokines enhanced the Tfh- and B-cell responses within the GC of the draining lymph nodes (31), although the precise mechanisms are not yet known and are the subject of ongoing studies. In this regard, it is suggested that future studies using a systems approach (34) to understand the induction of the numerous elements involved in the immune response to antigen processing within the lymph node microenvironment should prove extremely valuable to understanding vaccine efficacy.

## Data availability statement

The raw data supporting the conclusions of this article will be made available by the authors, without undue reservation.

## Ethics statement

The animal study was approved by Institutional Animal Care and Use Committee at the University of Utah. The study was conducted in accordance with the local legislation and institutional requirements.

## Author contributions

DG: Conceptualization, Funding acquisition, Investigation, Methodology, Project administration, Resources, Supervision, Writing – original draft, Writing – review & editing. JL: Investigation, Writing – review & editing. NN: Investigation, Writing – review & editing. FF: Conceptualization, Formal analysis, Supervision, Writing – review & editing. LH: Formal analysis, Investigation, Resources, Supervision, Writing – review & editing. RK: Formal analysis, Investigation, Writing – review & editing. Y-LC: Formal analysis, Investigation, Writing – review & editing. JH: Supervision, Writing – review & editing. JSH: Investigation, Supervision, Writing – review & editing. EDW: Formal analysis, Supervision, Writing – review & editing.
